# Parental acceptance of silver fluoride as a treatment option for carious lesions among South African children with special health care needs

**DOI:** 10.3389/froh.2023.1294227

**Published:** 2023-11-16

**Authors:** N. Potgieter, N. Noordien, R. Mulder, C. Peck, S. Groisman

**Affiliations:** ^1^ Department of Orthodontics and Paediatric Dentistry, University of the Western Cape, Cape Town, South Africa; ^2^ Department of Prosthodontics, University of the Western Cape, Cape Town, South Africa; ^3^ Faculdade de Odontologia, Universidade Federal do Rio de Janeiro, Rio de Janeiro, Brazil

**Keywords:** parental acceptance, silver fluoride, silver diamine fluoride, special care dentistry, caries

## Abstract

Providing dental care for children with neurological special health care needs, including Down syndrome, Cerebral palsy and Autism spectrum disorders, is challenging. They often require repeat exposure to sedation or general anaesthesia for routine dental care. 51 parents of children with special needs completed a questionnaire regarding the acceptance of Silver Fluoride as a treatment option.

**Background:**

Silver Diamine Fluoride has become popular as a minimally invasive treatment option for providing oral health care to young or uncooperative children. Silver Fluoride (SF) is a newer development with similar but improved properties. The aim was to determine the acceptance of SDF/SF as treatment option for Children with Special Health Care Needs (CSHCN), including Down Syndrome, Autism Spectrum Disorder and Cerebral Palsy.

**Methods:**

51 Parents of CSHCN completed a questionnaire on the overall acceptance of SF; aesthetic concerns related to the location of application; the use of SDF as an alternative to general anesthesia; and the composition of SF.

**Results:**

The use of SF on posterior teeth were more acceptable (70.59%) as opposed to its application to anterior teeth (50.98%). Parents generally agreed/ strongly agreed to the use of SF to reduce infection and pain (82%); to avoid treatment under GA (26.70%); and to avoid an injection (78%). 64% of parents indicated their agreement in using SF because it has a reduced cost when compared to a conventional restoration. Majority of parents were in agreement to use SF even if it contains Fluoride (84%) and Silver (78%).

**Conclusion:**

The use of SF, as treatment option for caries, was well accepted by South African parents of CSHCN. Shared decision making should be applied when considering SF as treatment option for CSHCN.

## 1. Introduction

Within the group of patients with special health care needs are three very common neurological conditions namely Cerebral Paralysis (CP), Down Syndrome (DS) and Autism Spectrum Disorder (ASD). These conditions not only affect the paediatric patients' general health but also their craniofacial development and subsequent oral health (1).

Several studies have highlighted the difficulties of performing dental care on children with a serious mental disability like CP, DS and ASD (1–3). Non-pharmacological behavior management of children with neurological conditions is often challenging as they often struggle with communication and social interaction. Linking with patient cooperation, restoration of cavities requires cooperation, time and moisture control which is often not possible in children with CP, DS and ASD. The Atraumatic Restorative Technique can be considered as a treatment option, however, still requires moderate cooperation to execute and may therefore not be applicable to all patients. As a result, children with CP, DS and ASD often require sedation or general anesthesia to enable routine restorative dentistry procedures to be performed. The risks of exposing children with CP, DS and ASD to repeated sedation and general anesthesia for routine dental treatment is worrying, and hence alternative treatment options are needed.

Silver Diamine Fluoride (SDF) has emerged as one such an alternative arrest dental caries, while being non-invasive and clinically efficient (4). The new generation, ammonia free Silver Fluoride (SF) has the added benefits of less tissue burn and irritation, improved smell, and better stability. Both Silver Diamine Fluoride (Riva Star) and Silver Fluoride (Riva Aqua) is approved for medical use with product registration numbers (D349082/K172047/GMDN code 45232 Class iia).

Parental acceptance of SDF treatments among healthy children has been reported as a limitation, due the resulting unaesthetic black staining when applied to carious lesions (5–7). Limited scientific evidence is available regarding the acceptancy of these techniques among children with special health care needs and their parents.

## 2. Methods, results and discussion

### 2.1. Material and methods

The aim of this study was to determine the acceptance of special needs children's parents of SF as a treatment option for carious lesions. The quantitative cross-sectional study was conducted by means of a self-administered questionnaire. The questionnaire included several relevant constructs, namely the overall acceptance; aesthetics concerns by tooth location, and its use as an alternative treatment in order not to admit the children to general anaesthesia for dental treatment.

Parents of children with special health care needs, meeting the inclusion criteria, were invited to participate in this study. The inclusion criteria was parents of patients diagnosed with DS, CP or ASD, in need of dental treatment (at least having one cavity indicated for restoration). Parents were informed about the nature, benefits and risks associated with the study after which written consent for voluntary participation was obtained. After consent was obtained, the parents were educated about SF as alternative to dental restorations by means of an information leaflet, adapted from the British Society of Paediatric Dentistry's patient leaflet for SDF (BSPD) (8). Participants then completed the questionnaire (it was printed in English but a translator was available in Afrikaans and Xhosa when necessary).

A convenience sample of 100 participants, in line with a peer review article published by Bagher et al. (9) was used. From the 100 participants approached, 20 parents declined participation. Questionnaires that were incomplete or had double answers marked were excluded from the study resulting in *n* = 51 questionnaires that were analyzed, indicating a 51% response rate.

#### 2.1.1. Ethical considerations

Ethical clearance was obtained from the Biomedical Research Ethics Committee (BM22/7/9) of the University of the Western Cape. All parents who participated in the questionnaire were not obliged to take SF as a treatment option and could opt for conventional treatment as routinely offered by the hospital. Patients screened for the study that did not meet the inclusion criteria but still required dental treatment received routine care. Parents had the option to opt out from the research at any time with the option to receive conventional restorative treatment or no further treatment. All patients were advised to attend regular follow up visits, regardless of their participation in the study. In terms of the requirements of the Protection of Personal Information Act (Act 4 of 2013), personal information was collected and processed as explained in the information sheet and consent from participants to use this information in the study was obtained on the consent form.

### 2.2. Results

Fifty-one Questionnaires were available that were correctly completed by the parent/guardian. The participating parents represented 20 (39.21%) children with Autism, 22 (43.13%) with Cerebral Palsy and 9 with Down Syndrome 9 (17.64%). The demographics of the participating parents and their children are summarized in Table 1, with the majority of parents being female (80.39% and between the ages of 30–49 years (52.93%). The children of participating parents were mostly male (68.62%) and between the ages of 6–10 years.

**Table 1:** Demographics of participating parents and their children.

Variable	*N* (%)
Child age	
0–5 years	13 (24.49)
6–10 years	26 (50.98)
11–15 years	12 (23.52)
>16 years	0 (0)
Male	35 (68.62)
Female	16 (31.37)
Other	0
Parents	
Female	41 (80.39)
<20 years	0 (0)
20–30 years	13 (25.49)
30–40 years	21 (41.17)
>50 years	7 (13.72)
Male	10 (19.61)
<20 years	1 (1.96)
20–30 years	1 (7.96)
30–40 years	6 (11.76)
>50 years	2 (3.92)

The parental acceptance with regards to the use of silver fluoride is reported in Table 2. Majority of parents (*n* = 38, 74.5%) agreed/strongly agreed to the use of SF for their children. The use of SF on posterior teeth was more acceptable (*N* = 36, 70.59%) as opposed to its application to anterior teeth (*N* = 26, 50.98%). Parents generally agreed/ strongly agreed to the use of SF to reduce infection and pain (*N* = 42, 82%); to avoid treatment under GA (*N* = 26.70%); and to avoid an injection (*N* = 40, 78%). 64% of parents indicated their agreement in using SF because it has a reduced cost when compared to a conventional restoration. Majority of parents were in agreement to use SF even if it contains Fluoride (*N* = 43, 84%) and Silver (*N* = 40, 78%).

**Table 2:** Parental acceptance of SF use reported in percentages.

Variable	Definitely disagree *N* (%)	Disagree *N* (%)	Neutral *N* (%)	Agree *N* (%)	Definitely agree *N* (%)
Use of SF on front teeth	9 (17.64)	9 (17.64)	7 (13.72)	17 (33.33)	9 (17.64)
Use of SF on back teeth	6 (11.76)	4 (7.84)	5 (9.80)	26 (50.98)	10 (19.60)
Use to reduce infection and pain	3 (5.88)	3 (5.88)	3 (5.88)	25 (49.01)	17 (33.33)
Use to avoid treatment under GA	3 (5.88)	7 (13.73)	4 (7.84)	26 (50.98)	11 (21.57)
Use to avoid injection	2 (3.92)	6 (11.76)	3 (5.88)	27 (52.94)	13 (25.49)
Use for reduced cost compared to filling	4 (7.84)	9 (17.65)	5 (9.80)	26 (50.98)	7 (13.72)
Use even if it contains fluoride	2 (3.92)	2 (3.92)	4 (7.84)	35 (68.62)	8 (15.68)
Use even if it contains silver	2 (3.92)	3 (5.88)	5 (9.80)	34 (66.66)	6 (11.76)
Overall acceptance of use	4 (7.84)	4 (7.84)	5 (9.80)	29 (56.86)	9 (17.65)

The result of the chi-square statistical analysis is reported in Table 3. The Chi-square statistic of 15.7669 is not statistically significant at *p* = 0.469343.

**Table 3:** Chi-square analysis of results.

	Results					
	SF front teeth	SF back teeth	Prevent GA	Avoid injection	SF accepted to use	Row totals
Definitely disagree	9 (4.80) [3.68]	6 (4.80) [0.30]	3 (4.80) [0.68]	2 (4.80) [1.63]	4 (4.80) [0.13]	24
Disagree	9 (6.00) [1.50]	4 (6.00) [0.67]	7 (6.00) [0.17]	6 (6.00) [0.00]	4 (6.00) [0.67]	30
Neutral	7 (4.80) [1.01]	5 (4.80) [0.01]	4 (4.80) [0.13]	3 (4.80) [0.68]	5 (4.80) [0.01]	24
Agree	17 (25.00) [2.56]	26 (25.00) [0.04]	26 (25.00) [0.04]	27 (25.00) [0.16]	29 (25.00) [0.64]	125
Definitely agree	9 (10.40) [0.19]	10 (10.40) [0.02]	11 (10.40) [0.03]	13 (10.40) [0.65]	9 (10.40) [0.19]	52
Column totals	51	51	51	51	51	255

### 2.3. Discussion

Although SDF if widely researched, the newer Silver Fluoride is not. Most of the comparisons and agreements will be drawn with parental acceptance studies on SDF. However, over time our hypothesis is that acceptance will only improve due to the newer properties of SF.

Bahger et al. (9) reported a 57.8% response rate from a similar questionnaire relating to children aged between 2 and 12 years with a mean ± SD age of 7.27 ± 2.35. These demographics are comparable with our study, at a 51% response rate with majority of the children being between the ages of 6 and 10 (50.98%). Majority of the children (whose parents participated in this study) were diagnosed with ASD and were male. This is in alignment with a higher incidence of ASD being diagnosed in males and the findings of Hu et al. (5) who reported 83% male participants in a SDF acceptance study among children diagnosed with ASD. Moreover, parents' level of acceptance towards SDF increased according to the level of increased difficulty that their child would experience in order to receive treatment (7).

An important factor which influences a parent's decision to accept the use of SDF/ SF in their children's mouth, is whether it is being applied anteriorly or posteriorly due to aesthetic concerns. The acceptance rate for anterior teeth was 50.97% (agree and strongly agree), while for posterior teeth was 70.58% (agree and strongly agree), which concurs with what is reported in the literature. Hu et al. (5) found the parental acceptance rate to increase from 35% for anterior teeth to approximately 67.5% for posterior teeth, while Crystal et al. (7) reported 29.7% and 67.5% respectively. Although no statistically significant difference could be found between the acceptance for use on anterior and posterior teeth in this study, Bahger et al. (9) reported significantly higher acceptance of SDF treatment on their child's primary compared to permanent teeth and posterior compared to anterior in both dentitions (*P* < 0.001).

However, Crystal et al. (7) further found that the acceptance rate for the use on anterior teeth increased to 60.3% when taking the risk of general anaesthesia into account, which echoes the finding of this study as 72.55% of parents supported the use of SDF to reduce to possibility of general anaesthesia for their child's treatment. Similarly, Hu et al. (5) reported an increase from 60% to 70% when the reality of general anaesthesia was given as a path to facilitate dental treatment and that parents of younger children are more likely to accept SDF as an alternative to GA.

The main ingredients of SDF are fluoride and silver, both of which have been elicited controversy in the past with regards to its safety for use in oral health care. The results from this study indicate that 84.3% of parents did not object to the product containing fluoride or silver (78.42%) for the combined “agree” and “strongly agree” Likert scales. Hu et al. (5) reported that more than 60% of parents would use SDF despite it containing silver and fluoride.

Children with ASD have higher levels of dental fear (5), which poses real contextual difficulties in receiving and delivering optimal dental care. This can delay treatment and increase dental pain, often further complicating the effective treatment pathways. In this study, 82.34% of parents accepted the use of SDF for the possibility of it reducing dental pain, avoiding intra-oral injections (78.43%) and possibly reducing dental costs of restorations (64.7%) as opposed to a minimal approach such as SDF. Both dental pain of pathological origin and giving injections feed dental fear. Crystal et al. (7) reported an increased level of acceptance of SDF as the level of difficulty that their child would experience in order to receive treatment increased, and parents of children with previous uncooperative behavior during dental treatment were significantly more accepting of the use of SDF, regardless of the type (primary or permanent) and location of the teeth (9).

Furthermore, Bagher et al. (9) found no statistically significant difference between the parental acceptance rate of SDF usage with the child's gender, parent's gender, parental education level and family income, which illustrates that the demands of special needs children are more influential on parental decision making (such as pain, anxiety, fear, communication challenges and need for treatment under GA). As such, the overall acceptance of SDF usage in this study was 82.34% (for both “agree” and “strongly agree” scales), which is somewhat higher than was reported by Hu et al. (5), as 60% in Singapore. Given that the dental demands of a developing country such as South Africa would be vastly different from that of a developed country like Singapore, this is not surprising. The burden of disease, caries prevalence and lack of appropriate dental care services for special needs children, makes the benefits of SDF very appealing to families who are struggling to meet the complex dental needs of their children. This not only includes financial constraints, but logistic challenges to find care to promote and maintain their children's oral health without compounding dental fears (i.e., with injections, fillings, extractions, etc.) or having no other options than being referred to GA for extensive treatment plans. It is clear, from this study and international literature, that parental acceptance with the use of SDF, despite its unaesthetic outcome, greatly increases when the benefits are weighed against the risks of not using it, given what the more extensive options are of not arresting active caries early.

## 3. Conclusion

SF, as treatment option for caries, was well accepted by South African parents of CSHCN and should be offered as a treatment option when indicated. Shared decision making should be applied when considering SF as treatment option for CSHCN.

### 3.1. Possible benefits of the study to the population

SF application in carious lesions is economic (cost and time effective) and safe not requiring local anaesthesia, sedation or general anaesthesia. The results of this study indicate that majority of parents with CSHCN would accept SF as a treatment option. Dental professionals should therefore consider and present SF as a treatment option for this vulnerable group of children.

## Data Availability

The raw data supporting the conclusions of this article will be made available by the authors, without undue reservation.

## Supplementary material

ANNEXURE I

Patient information sheet, SF information and consent form.

ANNEXURE II

Questionnaire.
