# Supplementary material for: Parental acceptance of silver fluoride as a treatment option for carious lesions among South African children with special health care needs
Source: Front Oral Health. 2023 Nov 16;4:1294227. doi: 10.3389/froh.2023.1294227 (PMC10687540; doi:10.3389/froh.2023.1294227)
Supplement: Supplementary file 1 [file Datasheet1.docx]

**Patient Information Sheet**


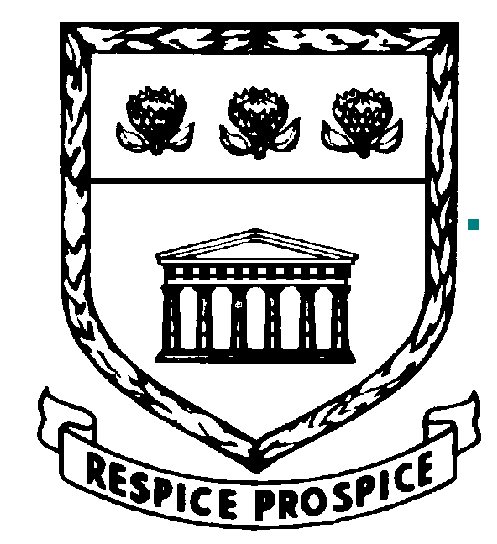

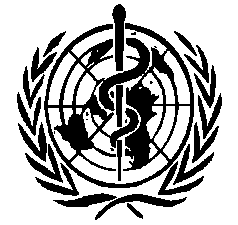


**Faculty of Dentistry & WHO Collaborating**

**Centre for Oral Health**

**UNIVERSITY OF THE WESTERN CAPE**

Private Bag X1, Tygerberg, Cape Town

REPUBLIC OF SOUTH AFRICA

**Project Title:** Parental acceptance of the use of Silver Fluoride (Riva Aqua) as treatment option for carious lesions in children with special health care needs

**What is this study about?**

This research aims to determine the acceptance of special needs children’s parents/ legal guardians of Silver Fluoride (SF) as a treatment option for carious lesions (holes in the teeth). SF is registered medical product and already used in dental clinics across South Africa.

**What will I be asked to do if I agree to participate?**

You will be asked to sign a consent form agreeing to take part in the study. Your participation will involve reading an information leaflet on SF as a treatment option for holes in the teeth and then completing a questionnaire on the overall acceptance, aesthetic concerns by tooth location, its use as an alternative treatment in order not to submit the children to general anaesthesia for dental treatment.

**Would my participation in this study be kept confidential?**

Your name will only be visible on the consent form and will be kept strictly confidential. The consent form and questionnaire will be numberred and only the principle researcher will be able to link a quesitonnaire with the consent form. The quesionnaire will ask your child’s age, child’s sex, child’s medical condition, parent’s age and parent’s sex. Original data (consent forms and questionnaires) will be kept for 5 years and and thereafter will be destroyed. Data sets without participant names will be preserved online and be accessible for future research.All requirements for the POPI Act will be adhered to and the data sets or resulting publications will not contain any names of participants. No names will be shared with third parties.

**What are the risks of this research?**

There are minimal or no foreseeable risks associated with participation. As described above, all precautions (coding of data, restricted access, storage in locked cabinets and/or password-protected computers) to protect anonymity and identity will be strictly applied.

**Why is this research important?**

This research is important because it will help in determination the parental acceptance of SF as treatment for children with special health care needs. The anonymized data from this study will be preserved and be accessible for future research.

**Do I have to be in this research and may I stop participating at any time?**

Your participation in this research is completely voluntary. You may choose not to take part at all. If you decide to participate, you have the right to withdraw at any time.If you decide not to participate in this study or if you stop participating at any time, you will not be penalized in any way.

**Is any assistance available if I am negatively affected by participating in this study?**

If at any time of the study, you feel uncomfortable and need assistance, the researcher will refer you for counselling through the social welfare office in your area.

**What if I have questions?**

Should you have any questions regarding this study and your rights as a research participant or if you wish to report any problems you have experienced related to the study, please do not hesitate to contact:

**Researcher: Dr N Potgieter**

Paediatric Dentistry, Faculty of Dentistry, University of the Western Cape

Tel: +27 21 937 3107 Email: nipotgieter@uwc.ac.za

**Research Ethics Committee**

Biomedical Research Ethics Committee (BMREC), University of the Western Cap

Private Bag x17, Bellville, 753

Tel: +27 21 959 411 Email: research-ethics@uwc.ac.za

**Information provided to parents for SF as treatment option for their child**


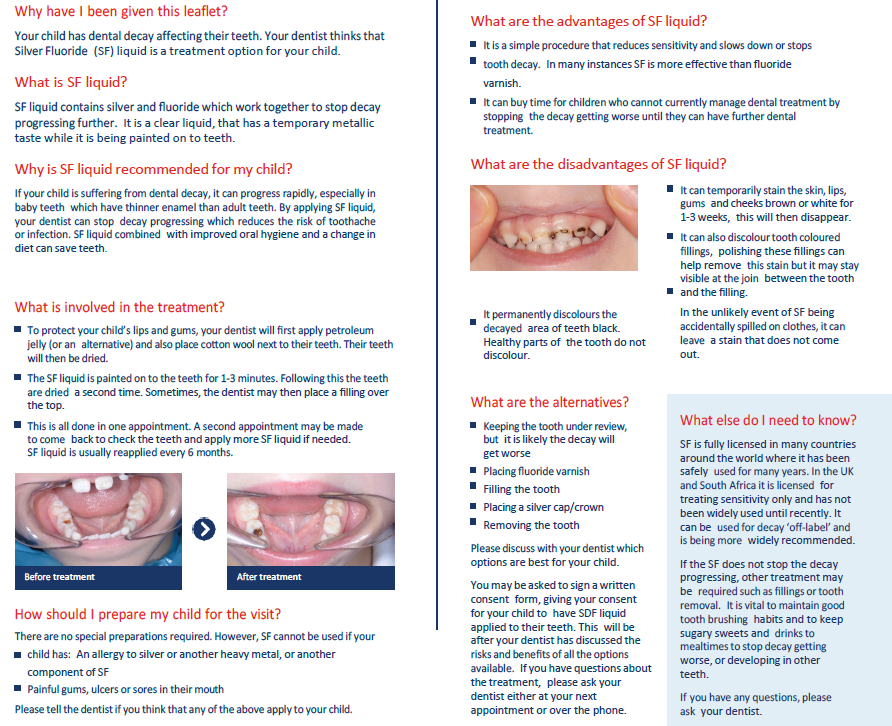


Adapted from: [**https://www.bspd.co.uk/Portals/0/BSPD_SDF%20patient%20leaflet_For%20website.pdf**](https://www.bspd.co.uk/Portals/0/BSPD_SDF%20patient%20leaflet_For%20website.pdf)

**Consent Form**


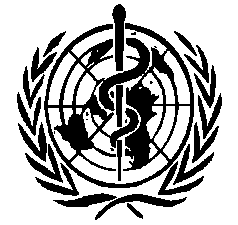


**Faculty of Dentistry & WHO Collaborating**

**Centre for Oral Health**


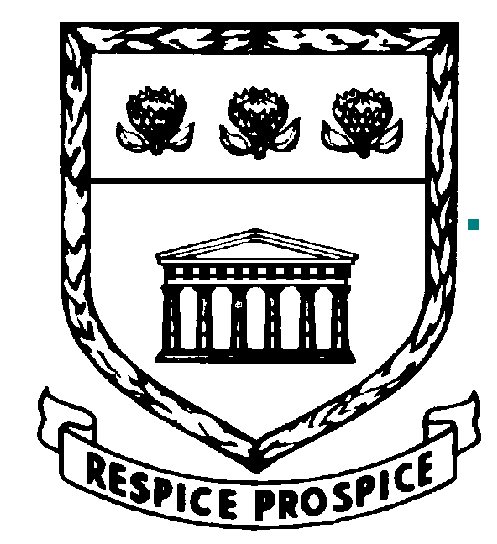


**UNIVERSITY OF THE WESTERN CAPE**

Private Bag X1, Tygerberg, Cape Town

REPUBLIC OF SOUTH AFRICA

**Project Title:** Parental acceptance of the use of Silver Fluoride (Riva Aqua) as treatment option for carious lesions in children with special health care needs

We invite you to participate as a volunteer in the study.

**RESEARCH INFORMATION:**

This research aims to determine the acceptance of special needs children’s parents/ legal guardians of Silver Fluoride as a treatment option for carious lesions. For this proposal parents/ legal guardians will be asked to complete a questionnaire on the overall acceptance, aesthetic concerns by tooth location, its use as an alternative treatment in order not to submit the children to general anaesthesia for dental treatment. All data will be presented without any personal identification and the consent forms (containing names) will be kept safe under the responsibility Dr. Nicoline Potgieter for a period of 5 years.

|  |
| --- |

**Declaration by the Participant**

I (full name) ……………………………………………………………………………….

- Declare that the study has been described to me in language that I understand;
- Have read, understood and received a copy of the information sheet and consent form, written in a language with which I am fluent;
- Have had the opportunity to ask questions regarding the study and my questions have been answered to my satisfaction;
- I understand that my identity will not be disclosed and that I have the right to withdraw from the study at any stage without giving a reason and without the risk of penalty; and that it will not negatively affect me in any way.

Do you freely and voluntarily choose to participate in this study?

Yes

No

In terms of the requirements of the Protection of Personal Information Act (Act 4 of 2013), personal information will be collected and processed as explained in the information sheet:

☐  I hereby give consent for my personal information to be collected, stored, processed and shared as described in the information sheet.

☐  I do not give consent for my personal information to be collected, stored, processed and shared as described in the information sheet.

_________________ __________________ _________________

Participant signature Researcher signature Date
