# Supplementary material for: Parental acceptance of silver fluoride as a treatment option for carious lesions among South African children with special health care needs
Source: Front Oral Health. 2023 Nov 16;4:1294227. doi: 10.3389/froh.2023.1294227 (PMC10687540; doi:10.3389/froh.2023.1294227)
Supplement: Supplementary file 2 [file Datasheet2.docx]

**Questionnaire:** Parental acceptance of the use of Silver Fluoride (Riva Aqua) as treatment option for carious lesions in children with special health care needs

**Demographics: Please tick the appropriate box:**

1. Age of the child patient:

| 0-5 years | 6-10 years | 11-15 years | >16 years |
| --- | --- | --- | --- |
|  |  |  |  |

1. Medical condition of the child patient:

| Autism | Cerebral Palsy | Down Syndrome |
| --- | --- | --- |
|  |  |  |

1. Sex of the patient:

| Male | Female | Other |
| --- | --- | --- |
|  |  |  |

1. Age of the parent:

| <20 years | 20-30 years | 30-40 years | >50 years |
| --- | --- | --- | --- |
|  |  |  |  |

1. Sex of the parent:

| Male | Female | Other |
| --- | --- | --- |
|  |  |  |

**Silver Fluoride as treatment option:**

1-I would use **Silver Fluoride** on my child to treat front teeth even if it turns teeth dark until it falls off.

| Definitely disagree | Disagree | Neutral | Agree | Definitely  agree |
| --- | --- | --- | --- | --- |
|  |  |  |  |  |

2-I would use **Silver Fluoride** on my child to treat back teeth even if it turns teeth dark until it falls off

| Definitely disagree | Disagree | Neutral | Agree | Definitely  agree |
| --- | --- | --- | --- | --- |
|  |  |  |  |  |

3- I would use **Silver Fluoride** on my child if it can reduce the chances of infection and pain

| Definitely disagree | Disagree | Neutral | Agree | Definitely  agree |
| --- | --- | --- | --- | --- |
|  |  |  |  |  |

4- I would use **Silver Fluoride** on my child if it means avoiding treatment under general anaesthesia

| Definitely disagree | Disagree | Neutral | Agree | Definitely  agree |
| --- | --- | --- | --- | --- |
|  |  |  |  |  |

5- I would use **Silver Fluoride** if my child can avoid having an injection

| Definitely disagree | Disagree | Neutral | Agree | Definitely  agree |
| --- | --- | --- | --- | --- |
|  |  |  |  |  |

6-I would use **Silver Fluoride** if it reduces cost of treatment when compared to traditional fillings

| Definitely disagree | Disagree | Neutral | Agree | Definitely  agree |
| --- | --- | --- | --- | --- |
|  |  |  |  |  |

7-I would use **Silver Fluoride** on my child even though it contains silver (within safety limits)

| Definitely disagree | Disagree | Neutral | Agree | Definitely  agree |
| --- | --- | --- | --- | --- |
|  |  |  |  |  |

8-I would use **Silver Fluoride** on my child even though it contains fluoride (within safety limits)

| Definitely disagree | Disagree | Neutral | Agree | Definitely  agree |
| --- | --- | --- | --- | --- |
|  |  |  |  |  |

9- I would use **Silver Fluoride** on my child

| Definitely disagree | Disagree | Neutral | Agree | Definitely  agree |
| --- | --- | --- | --- | --- |
|  |  |  |  |  |
